# A universal recombinant adenovirus type 5 vector-based COVID-19 vaccine

**DOI:** 10.3389/fimmu.2024.1374486

**Published:** 2024-04-30

**Authors:** Xingxing Li, Qinhua Peng, Xinyu Liu, Hongshan Xu, Jingjing Liu, Xiaohong Wu, Qiang Ye, Min Li, Yuhua Li

**Affiliations:** ^1^ Department of Arboviral Vaccine, National Institutes for Food and Drug Control, Beijing, China; ^2^ Office of Pharmaceutical Science of Biological Products, Center for Drug Evaluation, National Medical Products Administration (NMPA), Beijing, China

**Keywords:** adenovirus type-5, vaccination route, universal COVID-19 vaccine, broad-spectrum, SARS-COV-2 variants

## Abstract

A universal recombinant adenovirus type-5 (Ad5) vaccine against COVID19 (Ad-US) was constructed, and immunogenicity and broad-spectrum of Ad5-US were evaluated with both intranasal and intramuscular immunization routes. The humoral immune response of Ad5-US in serum and bronchoalveolar lavage fluid were evaluated by the enzyme-linked immunosorbent assay (ELISA), recombinant vesicular stomatitis virus based pseudovirus neutralization assay, and angiotensin-converting enzyme-2 (ACE2) -binding inhibition assay. The cellular immune response and Th1/Th2 biased immune response of Ad5-US were evaluated by the IFN-γ ELISpot assay, intracellular cytokine staining, and Meso Scale Discovery (MSD) profiling of Th1/Th2 cytokines. Intramuscular priming followed by an intranasal booster with Ad5-US elicited the broad-spectrum and high levels of IgG, IgA, pseudovirus neutralizing antibody (PNAb), and Th1-skewing of the T-cell response. Overall, the adenovirus type-5 vectored universal SARS-CoV-2 vaccine Ad5-US was successfully constructed, and Ad5-US was highly immunogenic and broad spectrum. Intramuscular priming followed by an intranasal booster with Ad5-US induced the high and broad spectrum systemic immune responses and local mucosal immune responses.

## 1 Introduction

According to the World Health Organization, as of March 15, 2024, over 774 million COVID-19 cases and almost 7 million related deaths had been reported globally (1). A total of 13.59 billion doses of COVID-19 vaccines have been reportedly administered globally, leading to a vaccination rate of 69.7% (2). At present, at least 24 types of COVID-19 vaccines have been authorized for emergency use in various countries. According to the list of global COVID-19 candidate vaccines published on the WHO website, a total of 183 candidate vaccines are in clinical development and 199 candidate vaccines are in the preclinical evaluation stage as of April 8, 2023 (3). Recombinant protein subunit vaccines account for the greatest proportion of vaccines in clinical development (59 vaccines, 32%), followed by RNA vaccines (43 vaccines, 24%). The other major vaccine types include inactivated virus vaccines (22 vaccines), viral vector (non-replicating) vaccines (25 vaccines), and DNA vaccines (17 vaccines) (3). Less common vaccine types include virus-like particle vaccines (7 vaccines), viral vector (replicating) vaccines (4 vaccines), and live attenuated virus vaccines (2 vaccines) (3). In China, a total of 15 COVID-19 vaccines have obtained conditional marketing authorization or approval for emergency use from the National Medical Products Administration (NMPA). These include 5 inactivated vaccines, 6 recombinant protein vaccines, 2 adenovirus-vectored vaccines, 1 influenza virus-vectored vaccine, and 1 mRNA vaccine. Among these 15 vaccines, 13 are administered intramuscularly and 2 are administered by inhalation or nasal spray.

The severe acute respiratory syndrome coronavirus 2 (SARS-CoV-2) is an enveloped positive-sense single-stranded RNA virus belonging to the order Nidovirales, family Coronaviridae, and genus Betacoronavirus (4). The SARS-CoV-2 genome encodes three major types of proteins: non-structural, structural, and accessory proteins. Non-structural proteins (NSPs) are primarily enzymes involved in viral replication processes. For instance, the genome encodes an RNA-dependent polymerase complex (NSP7, 8, and 12), an RNA capping mechanism (NSP10, 13, 14, and 16), and proteases that cleave the viral polyprotein (5, 6). Structural proteins mainly include the spike (S), envelope (E), membrane (M), and nucleocapsid (N) proteins. S, E, and M proteins are embedded in the lipid envelope, and the primary function of the N protein is to package the single-stranded, 5′-capped positive strand viral genome RNA molecule (approximately 30 kb in length) into a ribonucleoprotein (RNP) complex (7). The N protein can modulate the antiviral responses of the host and may also exert regulatory effects on the viral life cycle (7). Accessory proteins encoded by the ORF3a, 6, 7a, 7b, and 8 genes do not participate directly in virus replication but possess functions that can cause disruption to host innate immune responses (8). The rapid mutation rate of SARS-CoV-2 has led to varying degrees of decline in the immune efficacy of many COVID-19 vaccines (9, 10). Current variants of concern (VOCs) primarily comprise Omicron subvariants including BA. 1-BA. 5, BA. 2. 75, BQ. 1, XBB. 1. 5, and BF. 7, which possess considerably enhanced immune evasion capabilities (11, 12). Data from various studies have indicated that certain key sites with a high frequency of mutation (e.g., 69-70del, L452R, N501Y, P681R, K417N, E484K, and D614G) are the main causes of immune evasion (13, 14). By analysing the similarities and differences of high-frequency mutation sites among various VOCs, we designed and selected 10 high-frequency mutation sites in all VOCs (del69/70, K417N, T478K, L452R, E484K, D614G, H655Y, P681H, A701V) for the introduction of mutations into the S-gene sequence of the original SARS-CoV-2 strain. Overall, we hope to create a universal COVID-19 vaccine to combat the continuous emergence of new SARS-CoV-2 variants.

Adenoviruses are widely used as gene delivery vectors in vaccine development for various infectious diseases (e.g., haemorrhagic fever, acquired immunodeficiency syndrome [AIDS], influenza, malaria, and COVID-19) due to their advantages of high immunogenicity, broad tissue tropism, ease of genetic manipulation, difficulty of genome integration into host cells, availability of multiple serotypes for selection, and ability to elicit strong humoral and cellular immune responses (15). Multiple vaccines that employ human adenovirus type 5 (HAd5) as a vector have demonstrated the induction of strong humoral and cellular immune responses in preclinical and clinical studies (16–18). However, the high seroprevalence of HAd5 in the Chinese population (74. 2%) (19) has led to high preexisting immunity, which weakens the efficacy of HAd5-vectored vaccines.

Most COVID-19 vaccines are administered by intramuscular injection, which causes lower levels of mucosal immune responses. Recent research has reported that compared with intramuscular injection, intranasal administration of adenovirus-vectored vaccines generates higher titres of neutralizing antibodies (NAbs) and provides greater protective efficacy (20). However, intranasal administration of adenovirus-vectored vaccines also results in slightly lower cellular immune responses (21). Previous studies have indicated that combined intramuscular and intranasal immunization could overcome this bottleneck in immunization and enhance the effectiveness of adenovirus-vectored vaccines (21).

In addition, the potent immune evasion of the XBB sublineage is of concern, resulting in variable reductions in antibody titres against this variant induced by existing vaccines or convalescent sera. In patients infected with BA. 2 and BA. 4/5, in those who received 4 doses of the original strain mRNA vaccine, and in those who received the fourth dose of the bivalent (WuHan-Hu-1+BA. 5) mRNA vaccine, neutralizing antibodies against BA. 2 and BA. 4/5 were reduced by 2. 9-7. 8-fold and 3. 7-14-fold, respectively, compared with D614G, however, neutralizing antibodies against XBB were reduced by 66- to 155-fold (22).

In the present study, we constructed a universal adenovirus type-5 (Ad5)-vectored vaccine (Ad5-US) by homologous recombination using the AdEasy system. Subsequently, the immunogenicity of Ad5-US administered by different immunization routes in BALB/c mice was evaluated. Overall, we aimed to provide reference data for future research on the design approaches and vaccination regimens of universal adenovirus-vectored COVID-19 vaccines.

## 2 Materials and methods

### 2.1 Cells, vaccines and animals

Vero cells (African green monkey kidney cells) were stored for use at the Division of Arboviral Vaccines, National Institutes for Food and Drug Control (NIFDC, China). The Ad5-vectored COVID-19 vaccine Ad5-US was designed and constructed by the Division of Arbovirus Vaccine. The full-length S gene region of the SARS-CoV-2 B. 1. 351 strain was selected for gene design. Mutations were introduced at 10 high-frequency mutation sites in the S gene region that were common across the current VOCs (del69/70, K417N, T478K, L452R, E484K, D614G, H655Y, P681H, and A701V) among B. 1. 1. 7, B. 1. 351, P. 1. B. 1. 617. 2, BA. 1, BA. 4/5 and B. 2. 75 variants, respectively. The wild-type signal peptide (SP) of the S gene region was substituted with a tissue-type plasminogen activator SP. Mutations were introduced to the furin cleavage sites (R682G, R683S, R685S), and two proline substitutions (K986P, V987P). The full-length spike gene with multiple mutation sites was constructed into the shuttle plasmid, and the recombinant adenovirus plasmid was obtained by homologous recombination with the adenovirus backbone plasmid in bacteria. The recombinant adenovirus plasmid was transfected into HEK293 cells to obtain recombinant adenovirus expressing spike protein. After cultivating and purifying the viruses, the purified COVID-19 vaccine Ad5-US were used to evaluate the immunogenicity in mice. Specific-pathogen-free female BALB/c mice, aged 6 weeks, were provided and housed by NIFDC. Experiments involving animals were approved by and carried out in accordance with the guidelines of the Institutional Experimental Animal Welfare and Ethics Committee of NIFDC. (protocol code: 2021 (B) 038; date of approval: _21 December 2018_).

### 2.2 Evaluation of the immunogenicity of Ad5-US

#### 2.2.1 Ad5-US immunization regimens

**Figure 1:**
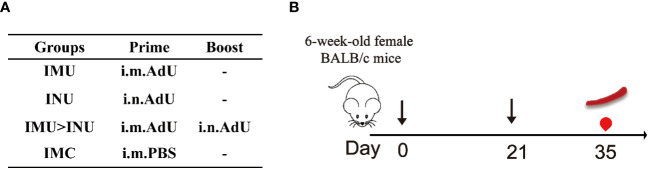
Mice immunization regimens. Prime, prime immunization; Boost, booster immunization; i.n., intranasal administration; i.m., intramuscular injection; IMU, intramuscular priming with Ad5-US and no booster immunization; INU, intranasal priming with Ad5-US and no booster immunization; IMU>INU, intramuscular priming followed by an intranasal booster with Ad5-US; IMC, intramuscular priming with PBS and no booster immunization; -, no booster immunization; 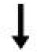, immunization; 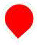, bleeding; 
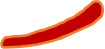
, spleen lymphocyte isolation.

**Figures 1A, B** show the overall protocol for animal grouping, immunization strategies, and immunological evaluation. The mice were randomly divided into four groups (n = 5) and immunized using different vaccine regimens: single-dose groups (1×INU, 1×IMU), two-dose groups with the same vaccination route (IMU > INU) and blank control groups (IMC). The time points for administration of the prime and booster doses were set as day 0 and day 21, respectively. The Ad5-vectored COVID-19 vaccine (Ad5-US) was administered in doses of 2×10^8 PFU per mice. On day 35, mice serum was collected, after which the mice were sacrificed and lymphocytes were separated from the spleen for further testing.

#### 2.2.2 Enzyme-linked immunosorbent assay

SARS-CoV-2 spike protein-specific immunoglobulin G (IgG) and immunoglobulin A (IgA) titres in serum and bronchoalveolar lavage fluid (BALF) were determined using ELISA. Serums were isolated from whole blood of mice after centrifugation. Bronchoalveolar lavage (BAL) of the lungs of mice was performed with 1 mL of 1× PBS, which yielded approximately 700 μL of BALF. Next, 0. 2 μg of S proteins (Sino Biological, Beijing, China) of the Wuhan-Hu-1, B. 1. 617. 2, and B. 1. 1. 529 strains were coated on a Costar ELISA 96-well plate (Corning, NY, USA) and incubated at 4°C overnight. The plate was subsequently washed with 1× PBST and blocked with 1× PBST containing 1% BSA at 37°C for 1 h. After the plate had been washed six times with 1× PBST, four-fold diluted serum was added to the wells. The plate was washed six times with 1× PBST again and incubated with either horseradish peroxidase-conjugated goat anti-mouse IgA (1:10,000; Abcam, UK) or goat anti-mouse IgG (1:10,000; ZSGB-BIO, China) at 37°C for 1 h. After washing, absorbance values at 450 nm and 630 nm were measured with TMB (3,3’,5,5’-tetramethylbenzidine; Beyotime, China) used as the substrate. IgG and IgA antibody titres in the serum were calculated using GraphPad Prism v9. The end point titres were defined as the highest reciprocal serum dilution, which was 2.1-fold higher compared to the negative control.

#### 2.2.3 Vesicular stomatitis virus pseudovirus neutralization assay

Mice serum samples were inactivated in a 56°C water bath for 30 min. The assay was performed in accordance with previously described methods (23). In short, serially diluted sera were separately mixed with 650 TCID50 (50% tissue culture infectious dose) of luciferase-expressing VSV-based Wuhan-Hu-1, Delta (B. 1. 617. 2), Omicron (B. 1. 1. 529), Omicron (BA. 2), Omicron (BF. 7), Omicron (BA. 2. 75), Omicron (BA. 2. 76), and Omicron (XBB) pseudoviruses and incubated in a 37°C incubator for 1 h. Vero cells (2 × 10^5 cells) were added, and the mixture was incubated at 37°C with 5% CO_2_ for 24 h. Relative luciferase activity was measured with a luciferase assay system (PerkinElmer, MA, USA). Cell control and virus control groups were established. Percent neutralization was calculated, and the half-maximal neutralization titres of the samples were calculated using the Reed–Muench method.

#### 2.2.4 ACE2 binding inhibition assay (ELISA)

V-PLEX COVID-19 ACE2 neutralization assay kits (Meso Scale Discovery, Panel 18 [ACE2] kit, K15570U; Panel 27 [ACE2] kit, K15609U-2; and Panel 29 [ACE2] kit, K15672U, Meso Scale Discovery, Rockville, MD, USA) were used for quantitative measurement of antibody levels that blocked the binding of ACE2 to its homologous ligands (including the S proteins of the Wuhan-Hu-1, B. 1. 1. 7, B. 1. 351, B. 1. 526. 1, B. 1. 617, B. 1. 617. 1, B. 1. 617. 2, B. 1. 617. 3, P. 1, P. 2, BA. 2, BA. 2. 12. 1, BA. 2. 75, BA. 2. 12. 1, BA. 2+L452M, BA. 2+L452R, BA. 3, BA. 4, and BA. 5 strains), i. e., the neutralization potency. The specific S protein antigen was coated onto a 96-well plate, and binding antibodies in the sample (diluted 1:100) were added. Detection was performed using the MSD SULFO-TAG-conjugated human ACE2 protein, and fluorescence emitted by the labels was measured on an MSD system. ACE2 binding inhibition=1-(average electrochemiluminescent signal of the sample/average electrochemiluminescent signal of the blank control) × 100%.

#### 2.2.5 IFN-γ ELISpot assay

Mice were sacrificed by cervical dislocation and immersed in 75% ethanol for 2–3 min. Next, 4 ~ 5 mL of lymphocyte separation medium (Dakewe, Beijing, China) was obtained, and spleen tissue was ground with a 2 mL syringe plunger and filtered through a 40 μm cell strainer. The splenocyte suspension was immediately transferred into a 15 mL centrifuge tube, overlaid with 1000 μL of RPMI 1640 medium (Hyclone, Cytiva, MA, USA), and centrifuged at 800 g and room temperature for 30 min. After centrifugation, the liquid in the 15 mL centrifuge tube was divided into four layers: (from top to bottom) RPMI 1640 medium overlay, lymphocyte layer, separation medium layer, and red blood cell and cell fragment layer. The lymphocyte layer was aspirated, and 10 mL of RPMI medium was added. Centrifugation was performed at 250 g and room temperature for 10 min, and the lymphocytes were collected. The supernatant was discarded, and cells were suspended in serum-free medium (Dakewe). IFN-γ-positive cells were detected using a mouse IFN-γ ELISpot assay kit (Mabtech, Stockholm, Sweden). A 96-well PVDF plate was washed four times with 200 µL of 1× PBS and blocked with RPMI-1640 medium containing 10% FBS at 25°C for at least 2 h. Freshly separated lymphocytes (2. 5 × 10^5 cells) were transferred into the wells and stimulated with S protein peptide pool(15-mers with 11-residue overlaps, 1 µg/mL per peptide, Genscript, Nanjing, China) of the Wuhan-Hu-1, B. 1. 617. 2, B. 1. 1. 529, and BA. 5 strains at 37°C for 24 h. After incubation with anti-mouse IFN-γ antibody for 2 h, the plates were incubated again with streptavidin-horseradish peroxidase (1:1,000 dilution, Dakewe) for 1 h. TMB solution (100 mL) was added into each well and developed for 5 min until the appearance of different spots. The ImmunoSpot^®^ S6 Universal instrument (Cellular Technology Limited, USA) was used to observe and count the spots.

#### 2.2.6 Intracellular cytokine staining

**Figure 2:**
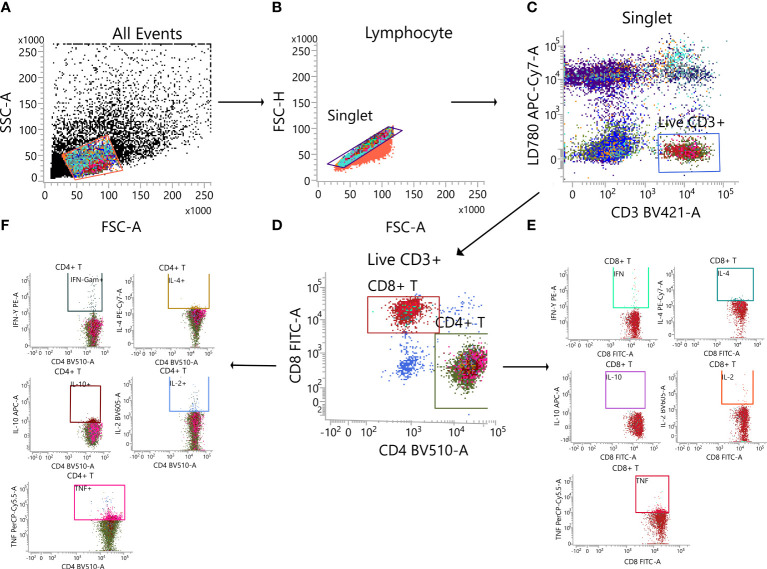
Gating strategy. **(A)** Lymphocytes. **(B)** Single cell. **(C)** Live CD3^+^ T cell. **(D)** CD4^+^T cell/CD8^+^ T cell. **(E)** Cytokine-secreting CD8^+^ T cell. **(F)** Cytokine-secreting CD4^+^ T cell.

Splenic lymphocytes were isolated and stimulated with 2 mg/mL of the spike protein peptide pool and brefeldin A (1:1,000 dilution, Biolegend, USA) at 37°C and 5% CO2 for 6 h. After stimulation, the splenocytes were rinsed and stained with fixable viability stain and 780 the following antibodies: BV421 hamster anti-mouse CD3e antibody, BV510 rat anti-mouse CD4 antibody, FITC rat anti-mouse CD8a antibody, and Fixable Viability Dye 780 (BD Biosciences, USA) to exclude dead cells. The splenic lymphocytes were washed twice with 1× PBS and fixed using Cytofix/Cytoperm (BD Biosciences). After washing the cells with 1× Perm/Wash buffer, staining was performed with PE-conjugated rat anti-mouse IFN-γ, BV605 rat anti-mouse interleukin (IL)-2, PE-Cy7 rat anti-mouse IL-4, APC rat anti-mouse IL-10, and BB700 rat anti-mouse tumour necrosis factor (TNF) (BD Biosciences). The cells were then washed with Perm/Wash buffer (BD Biosciences) and 1× PBS, resuspended in 1× PBS, and determined using a FACS Lyric flow cytometric analyser (BD Biosciences). For each sample, 200,000 events were recorded, and data analysis was performed with FlowJo software (TreeStar, USA). single cells were selected from the lymphocyte population (SSC-A vs. SSC-H), viable CD3^+^ T cells were derived from single cells (CD3^+^ vs. LD780^-^), CD4^+^ T cells (CD4^+^ vs. CD8^-^) and CD8^+^ T cells (CD8^+^ vs. CD4^-^), both of which were derived from viable CD3^+^ T cells. Respectively from the CD4^+^ T cells (CD4^+^ vs. IFN-γ^+^/IL-2^+^/IL-4^+^/IL-10^+^/TNF^+^) and CD8^+^ T cells (CD8^+^ vs. IFN-γ^+/^IL-2 ^+^/IL-4^+^/IL-10^+^/TNF^+^). Detection data are represented as the percentage of cytokine-positive cells in CD8^+^ or CD4^+^ T cells (**Figure 2**).

#### 2.2.7 Meso scale discovery cytokine assay

On Day 35 after priming, splenic lymphocytes were separated and stimulated for 24 h with peptide pool covering the S proteins of the Wuhan-Hu-1, B. 1. 617. 2, and B. 1. 1. 529 strains (15-mers with 11-residue overlaps, 1 µg/mL per peptide). The supernatant was collected, and the V-PLEX multiplex assay kit (mouse) was used for the measurement of IFN-γ, TNF-α, IL-2, IL-4, and IL-10 concentrations. Cytokine levels were measured using the MESO QuickPlex SQ 120 instrument (Meso Scale Diagnostics), and concentrations were calculated using the standard curve.

### 2.3 Statistical analysis

**Figure 3:**
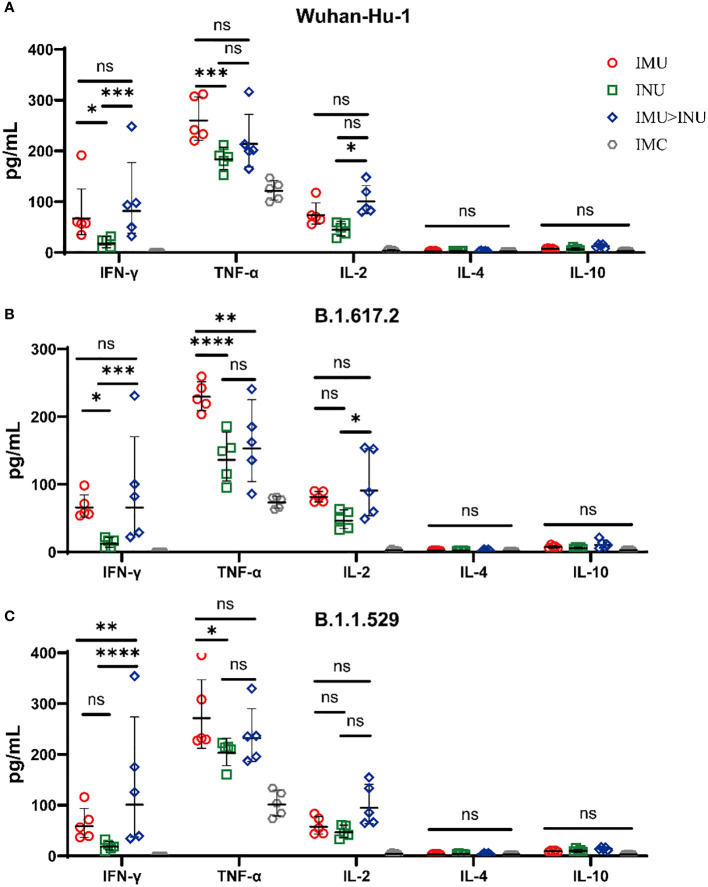
Determination of the Th1/Th2 bias of cellular immunity in mice immunized with Ad5-US using different immunization regimens by MSD cytokine analysis. On day 35 after priming, splenic lymphocytes were separated and stimulated for 24 h with peptide pool covering the S proteins of the entire **(A)** Wuhan-Hu-1, **(B)** B. 1. 617. 2, and **(C)** B. 1. 1. 529 strains. The supernatants were separately collected and subjected to measurement of IFN-γ, IL-2, TNF-α, IL-4, and IL-10 concentration levels (N=5 in each group, with one point representing one sample). ns: p > 0. 05, *: p < 0. 05, **p < 0. 01, ***p < 0. 001, ****p < 0. 0001. IL, interleukin; TNF, tumour necrosis factor; MSD, Meso Scale Discovery.

GraphPad Prism v9 software was employed for all plotting and statistical tests Data are shown as the geometric mean ± geometric standard deviation (except for ACE2-binding inhibition rates that are shown as mean ± standard deviation). We performed one-way or two-way analyses of variance only on **Figure 3** to analyse statistical differences among multiple groups (ns: *p* > 0. 05, *: *p* < 0. 05, **: *p* < 0. 01, ***: *p* < 0. 001, ****: *p* < 0. 0001).

## 3 Results

### 3.1 Combined intramuscular and intranasal vaccination with Ad5-US elicited high, broad-spectrum humoral immune responses

**Figure 4:**
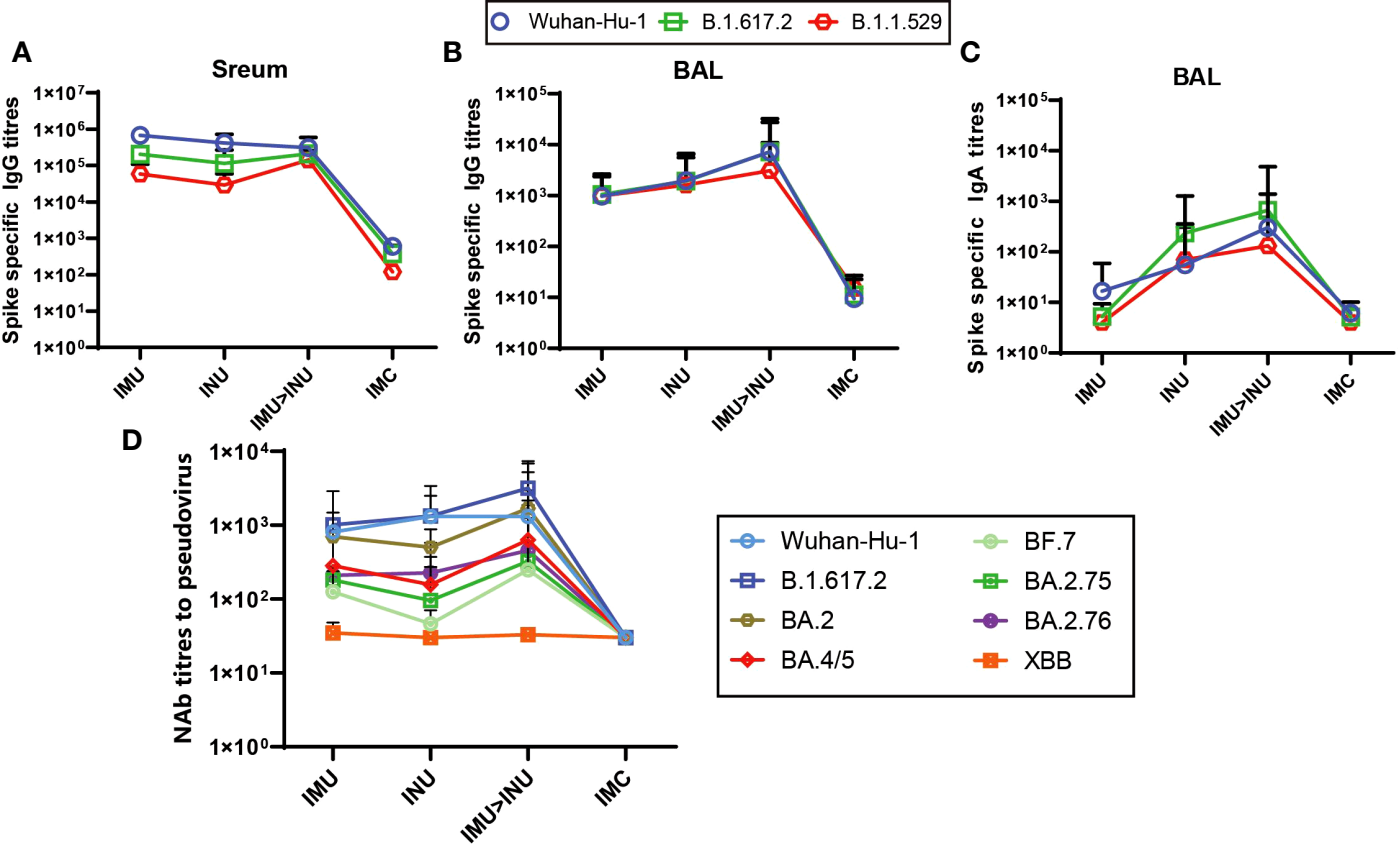
Humoral immune responses elicited by the Ad-US vaccine in different vaccination regimens. **(A)** Spike protein-specific binding IgG titres in serum; **(B)** Spike protein-specific binding IgG titres in BALF; **(C)** Spike protein-specific binding IgA titres in BALF; **(D)** NAb titres in serum. NAb titres are expressed as the EC50 (N=5 in each group). Ad5-US, Ad5-vectored COVID-19 vaccine; BALF, bronchoalveolar lavage fluid.

The spike-specific IgG titres (S-IgG) in both serum and BALF and spike-specific IgA titres (S-IgA) in the BALF on day 35 after priming were measured by ELISA (**Figures 4A–D**). As shown in **Figure 4A**, intramuscular priming followed by intranasal boosting group (IMU>INU) and the single-dose intranasal administration and single-dose intramuscular injection groups (INU, IMU) all developed high S-IgG levels against the Wuhan-Hu-1, B. 1. 617. 2, and B. 1. 1. 529 strains, the IMU>INU group against the Wuhan-Hu-1, B. 1. 617. 2, and B. 1. 1. 529 strains developed IgG geometric mean titres (GMTs) of 311809, 211664, and 143136, respectively. For all three strains, the S-IgG level elicited by the IMU group was slightly higher than that of the INU group.

In the BALF, the IMU>INU group elicited the highest S-IgG levels in BALF against the Wuhan-Hu-1, B. 1. 617. 2, and B. 1. 1. 529 strains (**Figure 4B**), with the GMTs being 7271, 7242, and 3089, respectively. For all three strains, the S-IgG level in BALF elicited by the INU group was slightly higher than that of the IMU group.

The measured S-IgA levels in BALF (**Figure 4C**) indicate that the IMU>INU group elicited the highest S-IgA levels against the Wuhan-Hu-1, B. 1. 617. 2, and B. 1. 1. 529 strains. The GMTs were 303, 664, and 131, respectively. S-IgA levels elicited by the INU group were slightly lower than those of the IMU>INU group. The IMU group did not elicit a significant mucosal immune response, with its S-IgA titres similar to those of the IMC group.

On day 35 after priming, the VSV pseudovirus assay was used to assess the specific NAb titres in serum against the original strain Wuhan-Hu-1, B. 1. 617. 2, and the Omicron variants (BA. 2, BA. 4/5, BF. 7, BA. 2. 75, BA. 2. 76) (**Figure 4D**). Among all vaccination groups, the IMU>INU group elicited the higher levels and more broad- spectrum of pseudovirus neutralizing antibodies (PNAbs) against Wuhan-Hu-1, B. 1. 617. 2, BA. 2, BA. 4/5, BF. 7, BA. 2. 75, and BA. 2. 76. The PNAb GMTs were 1318, 3178, 1688, 635, 248, 325, and 453, respectively. Levels of PNAbs elicited by the IMU>INU group against the various strains decreased in the following order: B. 1. 617. 2 > BA. 2 > Wuhan-Hu-1 > BA. 4/5 > BA. 2. 76 > BA. 2. 75 > BF. 7. Broad spectrum of the IMU and INU groups were slightly lower than those of the IMU>INU group. All Ad5-US immunization groups were nearly unsuccessful in eliciting PNAb levels against the XBB strain, which was comparable to the control IMC group.

**Figure 5:**
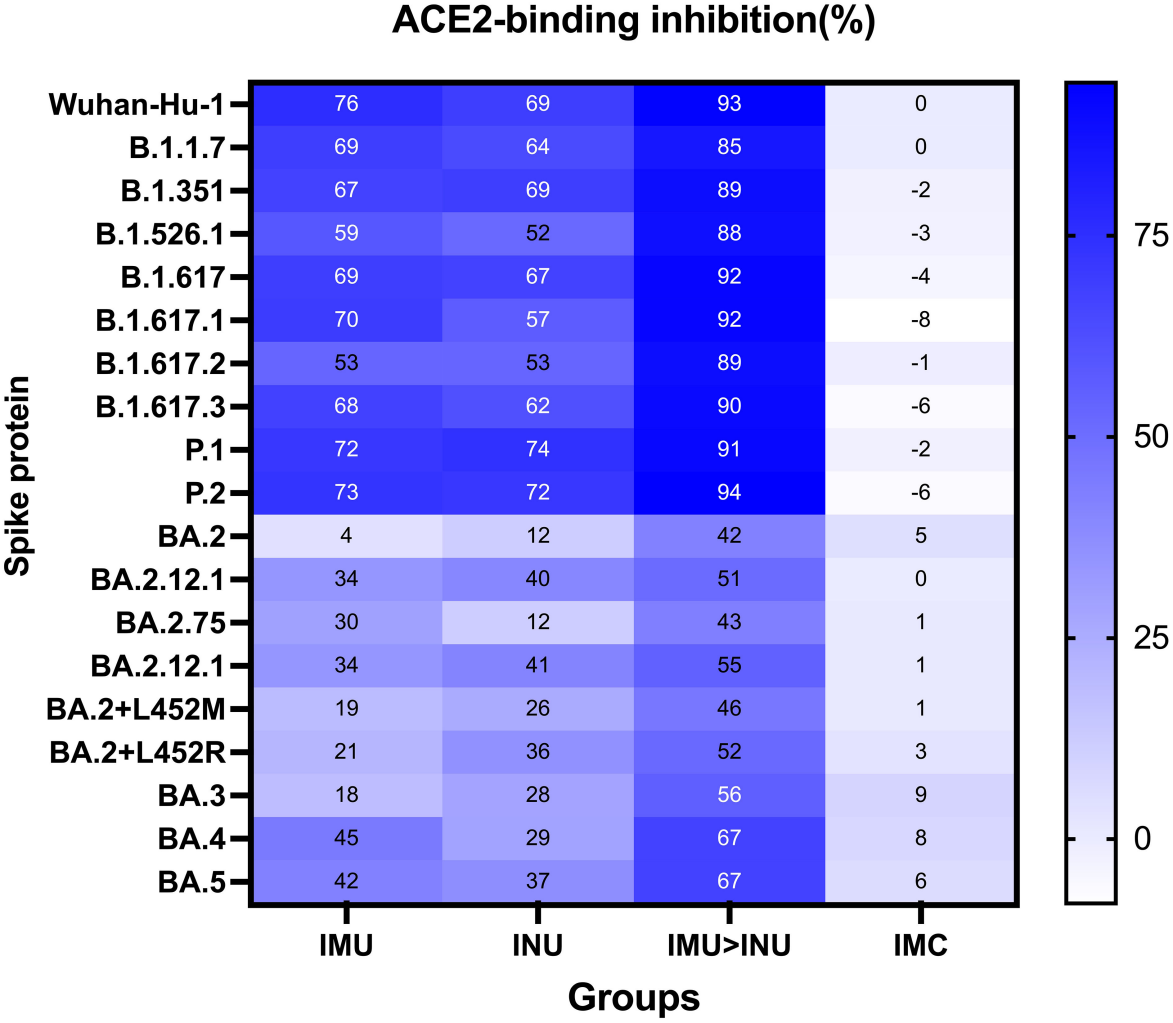
Neutralization capacity of sera was observed by measuring the inhibition of binding between angiotensin-converting enzyme 2 (ACE2) and SARS-CoV-2 spike proteins on day 35 after primary immunization. Spike proteins were from the SARS-CoV-2 prototype and B. 1. 1. 7, B. 1. 351, B. 1. 526. 1, B. 1. 617, B. 1. 617. 1, B. 1. 617. 2, B. 1. 617. 3, P. 1, P. 2, BA. 2, BA. 2. 12. 1, BA. 2. 75, BA. 2. 12. 1, BA. 2+L452M, BA. 2+L452R, BA. 3, BA. 4, and BA. 5 strains, respectively. Numbers indicate the average values of the corresponding groups (N=5 in each group).

The ACE2 binding inhibition assay was used to measure the ACE2 binding inhibition rates in the serum on day 35 after priming against the S protein of SARS CoV-2 variants (Wuhan-Hu-1, B. 1. 1. 7, B. 1. 351, B. 1. 526. 1, B. 1. 617, B. 1. 617. 1, B. 1. 617. 2, B. 1. 617. 3, P. 1, P. 2, BA. 2, BA. 2. 12. 1, BA. 2. 75, BA. 2. 12. 1, BA. 2+L452M, BA. 2+ L452R, BA. 3, BA. 4, and BA. 5) (**Figure 5**). Among all the vaccination groups, the IMU>INU group exhibited the highest levels and broadest spectrum of neutralizing activities, with the ACE2 binding inhibition rates against the Wuhan-Hu-1, B. 1. 1. 7, B. 1. 351, B. 1. 526. 1, B. 1. 617, B. 1. 617. 1, B. 1. 617. 2, B. 1. 617. 3, P. 1, P. 2, BA. 2, BA. 2. 12. 1, BA. 2. 75, BA. 2. 12. 1, BA. 2+L452M, BA. 2+L452R, BA. 3, BA. 4, and BA. 5 strains being 89%, 82%, 86%, 83%, 86%, 86%, 83%, 85%, 89%, 90%, 41%, 57%, 40%, 57%, 48%, 50%, 48%, 64%, and 66%, respectively. The cross-neutralizing activity levels elicited by the INU group (12–74%) were comparable to those of the IMU group (4–76%). Broad spectrum of the IMU and INU groups were slightly lower than those of the IMU>INU group. Which were comparable to the results of the PNAb assay.

### 3.2 All Ad5-US vaccination groups exhibited strong T-cell immune responses

**Figure 6:**
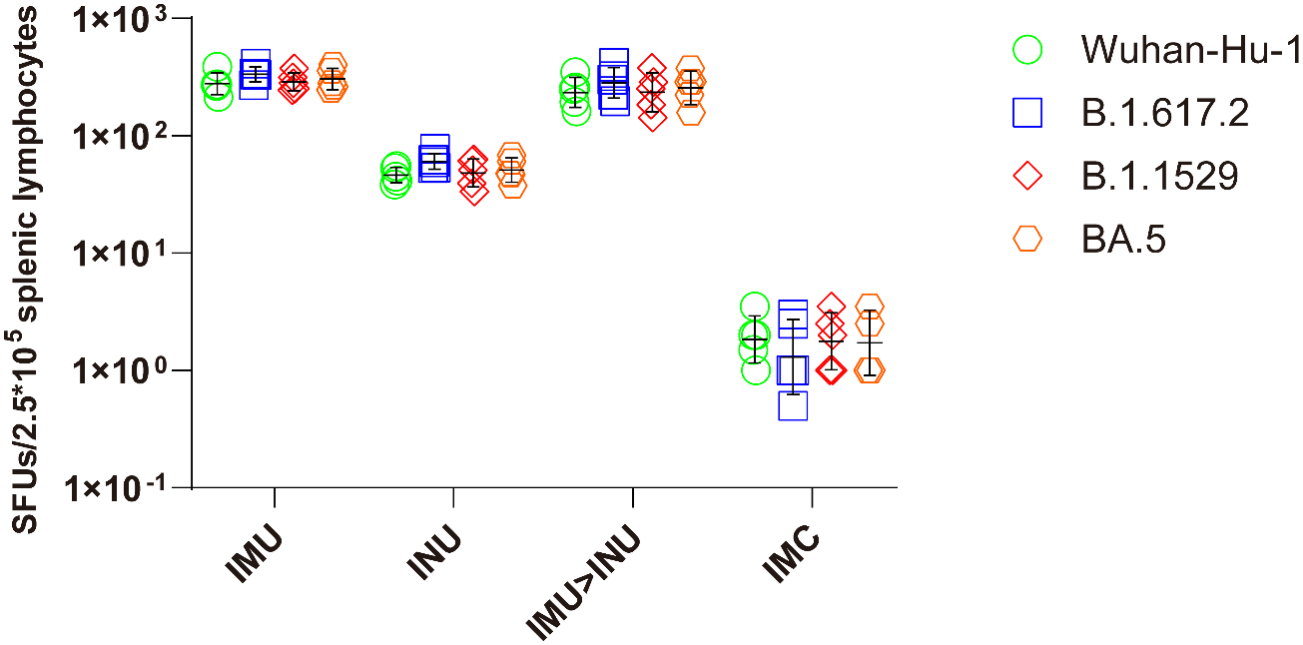
Measurement of S protein-specific cellular immune responses after vaccination with the Ad5-US vaccine via the ELISpot assay. On day 35 after priming, splenic lymphocytes were separated and stimulated with S protein peptide pool of Wuhan-Hu-1, B. 1. 617. 2, B. 1. 1. 529, and BA. 5. Cells secreting IFN-γ were quantitatively measured by the ELISpot assay (N=5 in each group, with each data point representing the average number of double spots for one sample).

On day 35 after priming, splenic lymphocytes were collected, stimulated with S protein peptide pool of Wuhan-Hu-1, B. 1. 617. 2, B. 1. 1. 529, and BA. 5 strains for 24 h, and subjected to the IFN-γ ELISpot assay (**Figure 6**). All vaccination groups demonstrated strong T-cell immune responses against the Wuhan-Hu-1, B. 1. 617. 2, B. 1. 1. 529, and BA. 5 strains. IFN-γ-positive spot GMTs against the Wuhan-Hu-1, B. 1. 617. 2, B. 1. 1. 529, and BA. 5 strains elicited by the IMU>INU group were 234, 282, 235, and 256, respectively. The T-cell immune responses elicited by the INU group were slightly lower than those of the IMU>INU and IMU groups, with the IFN-γ-positive spot GMTs against the Wuhan-Hu-1, B. 1. 617. 2, B. 1. 1. 529, and BA. 5 strains being 46, 61, 48, and 51, respectively.

### 3.3 Ad5-US vaccine elicited Th1-skewed cell immune responses

**Figure 7:**
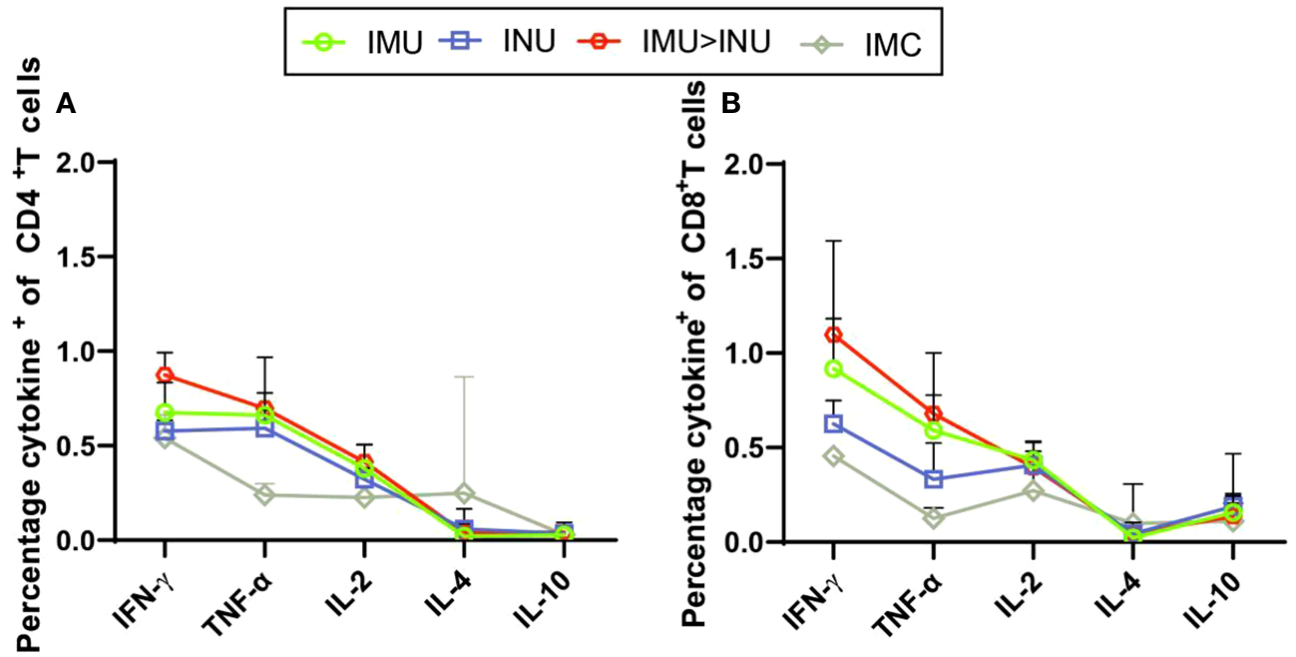
Assessment of the Th1/Th2 bias of T-cell immune responses in mice immunized with Ad5-US by intracellular cytokine staining. On day 35 after priming, splenic lymphocytes were separated and stimulated for 8 h with peptide pool covering the S proteins of the entire B. 1. 1. 529 strain. Percentages of CD4^+^ T cells **(A)** and CD8^+^ T cells **(B)** positive for IFN-γ, IL-2, TNF-α, IL-4, and IL-10 (N=5 in each group) were determined by intracellular cytokine staining. IL, interleukin; TNF, tumour necrosis factor.

Next, we evaluated the Th1 skewing of T-cell responses specific to spike proteins of B.1.1.529 variant. Splenic lymphocytes were harvested on day 35 after primary immunization and stimulated with the peptide pool. Intracellular cytokine staining showed a high percentage of splenic lymphocytes positive for hallmark Th1 cytokines IFN-γ, TNF-α, and IL-2 in all vaccination groups, but a low percentage of secreted typical cytokines (IL-4 and IL-10) associated with the Th2-type immune response (**Figures 7A, B**). Compared with the other groups, the IMU>INU group elicited the higher Th1-biased cellular immune levels.

MSD cytokine profiling assays (**Figures 3A–C**) showed that all Ad5-US immunization groups produced high concentrations and comparable levels of IFN-γ, TNF-α, and IL-2 against the Wuhan-Hu-1, B. 1. 617. 2, and B. 1. 1. 529 strains. By contrast, only low concentrations of IL-4 and IL-10 were detected, with differences with the control group being statistically insignificant. Compared with the INU group, the IMU>INU and IMU groups elicited higher levels of Th1-skewed cellular immune responses. These results indicate that the Ad5-US vaccine elicited Th1-biased cellular immune responses, with the intramuscular route being superior to the intranasal route.

## 4 Discussion

Currently, the immune evasion capabilities of various newly emerged COVID-19 variants have significantly increased, and the protective efficacy of multiple types of COVID-19 vaccines has shown varying degrees of decline (10, 24, 25). The development of a universal COVID-19 vaccine is, therefore, of great importance for addressing the immune evasion phenomenon of variant strains. In the present study, intramuscular priming followed by intranasal boosting of Ad5-US not only induced the high levels and broad-spectrums of IgG, IgA, and PNAb titres, but also elicited Th1-skewed cellular immune responses. this is a promising immunization strategy for adenovirus-vectored COVID-19 vaccines.

In my study, Ad5-US expressing 10 high-frequency mutation sites in the S gene region developed broad cross-NAb responses against multiple newly emerged variants. A possible reason for this phenomenon may be that these high-frequency mutation sites are closely associated with the antigenic epitope structure of the S protein. The changes in amino acid charge properties before and after the mutation may in turn affect the antigenic epitope structure of the S protein, thereby generating specific NAb responses against the newly emerged variant strains (26).

The subvariant XBB lineage resulting from recombination of BA2. 10. 1 with BA2. 75 was first identified in India. This lineage has relatively low pathogenicity, but it has strong transmissibility and high infection degree (22, 27). However, vaccination with Ad5-US hardly elicited significant levels of PNAbs against the XBB strain. A significant reduction in neutralizing antibody titres against the XBB lineage has been shown in persons who have received four doses of mRNA vaccine or who have had previous infections with BA. 2 and BA. 5 (22, 28–31). This may be related to the immune evasion mechanism caused by specific mutation sites in the XBB strain (27, 32). The XBB strain has a total of 22 mutation sites in the receptor binding domain (RBD) of the S gene, namely G339H, R346T, L368I, S371F, S373P, S375F, T376A, D405N, R408S, K417N, N440K, V445P, G446S, N460K, S477N, T478K, E484A, F486P, F490S, Q498R, N501Y, and Y505H. Compared with the BA. 5 strain, the XBB strain has six additional mutation sites (R346T, L368I, V445P, G446S, N460K, and F490S), while the L452R site remains unchanged. Studies have reported that five mutation sites (R346T, V445P, G446S, N460K, and F490S) affect NAb activity in the serum, thereby causing the phenomenon of immune evasion (12, 33, 34). The XBB 1. 5 lineage is the descendant of the XBB lineage, and the S468P mutation was added on the basis of the XBB mutation. This lineage has become the main circulating strain in many countries (29, 35, 36). According to the U. S. Centres for Disease Control and Prevention (CDC), XBB. 1. 5 accounted for 87. 9% of the circulating strains in the United States as of April 1, 2023 (37). Given the high transmission rate and high immune escape capacity of the XBB lineage, the FDA authorized and approved the monovalent XBB. 1. 5 mRNA vaccine from Pfizer-Biotech and Moderna on September 11, 2023, and the CDC recommended that persons older than 6 months receive the updated vaccine (38). And in a recent study, it was shown that a vaccine designed with the spike protein of XBB. 1. 5 could induce broad and strong immune protection against XBB. 1. 5 and other Omicron variants. However, vaccines based on Omicron variants with fewer mutations were less effective against XBB and XBB. 1. 5 (39). This coincides with our conclusion that in the face of mutants with more and more mutation sites, more mutation sites need to be introduced in the process of vaccine design to induce stronger immune protection.

Cellular and humoral immune responses complement each other. Therefore, effective cellular immune responses are of great significance for eliminating SARS-CoV-2, resisting invasion by various variants, and assisting B cells in eliciting multifunctional antibody responses (40). In the present study, all Ad5-US vaccination groups elicited Th1-skewed cellular immune responses on day 35 after priming. We also observed that Ad5-US vaccination induced comparable cellular immune response levels against the various SARS-CoV-2 variants, which demonstrates the induction of broad cross-reactive and multifunctional T-cell immune responses. Tarke et al. (41) found that memory T-cell responses induced by COVID-19 vaccines across different vaccine platforms (mRNA-1273, BNT162b2, Ad26. COV. S, and NVX-CoV2373) exhibited broad and comparable immunity levels against various SARS-CoV-2 variants. However, the levels of NAbs produced by memory B cells against newly emerged variants were significantly decreased, which is consistent with our research findings.

The present study has some limitations. First, Comparing the immune response levels of the one-dose and two-dose groups, it is imprecise and uncritical that we did not design the time of vaccination for the one-dose group to be the time of booster vaccination for the two-dose group. We will further refine the methodology and re-evaluate the immunization effects of this immunization strategy. Second, NAb titres against the live SARS-CoV-2 virus were not measured due to resource constraints. NAb levels are closely related to the protective efficacy of the vaccine. Thus, further virus challenge studies with SARS-CoV-2 and its variants will be required to demonstrate the broad-spectrum protective efficacy of the IMU>INU group. Third, there is an urgent need to assess T-cell immune levels against other newly emerged variants (e.g., BF. 7, JN.1.8, XBB. 1, and JN. 1) for a more comprehensive evaluation of the broad-spectrum cellular immune response levels of our proposed immunization strategy.

In conclusion, the homologous sequential immunization strategy of intramuscular priming followed by intranasal boosting with Ad5-US can induce strong and broad immune responses, which provides new approaches and reference data for future design of universal COVID-19 vaccines.

## Data availability statement

The raw data supporting the conclusions of this article will be made available by the authors, without undue reservation.

## Ethics statement

The animal studies were approved by Institutional Animal Care and Use Committee of the NIFDC. The studies were conducted in accordance with the local legislation and institutional requirements. Written informed consent was obtained from the owners for the participation of their animals in this study.

## Author contributions

XxL: Conceptualization, Writing – original draft. QP: Conceptualization, Writing – original draft. XyL: Methodology, Writing – original draft. HX: Software, Writing – original draft. JjL: Validation, Writing – original draft. XW: Formal Analysis, Writing – original draft. QY: Data curation, Resources, Supervision, Writing – original draft. ML: Project administration, Visualization, Writing – review & editing. YL: Project administration, Supervision, Visualization, Writing – review & editing.

## References

[1] World Health Organization. Weekly epidemiological update on COVID-19 . Available online at: https://www.who.int (Accessed 15 March 2024).

[2] World Health Organization. WHO coronavirus (COVID-19) dashboard . Available online at: https://covid19.who.int (Accessed 20 December 2023).

[3] World Health Organization. Tracking SARS-CoV-2 variants . Available online at: https://www.who.int (Accessed 19 April 2023).

[4] Fung TS Liu DX. Human coronavirus: host-pathogen interaction. Annu Rev Microbiol. (2019) 73:529–57. doi: 10.1146/annurev-micro-020518-115759 31226023

[5] Enjuanes L Almazán F Sola I Zuñiga S. Biochemical aspects of coronavirus replication and virus-host interaction. Annu Rev Microbiol. (2006) 60:211–30. doi: 10.1146/annurev.micro.60.080805.142157 16712436

[6] Lai MM. Coronavirus: organization, replication and expression of genome. Annu Rev Microbiol. (1990) 44:303–33. doi: 10.1146/annurev.micro.44.1.303 2252386

[7] Zúniga S Cruz JL Sola I Mateos-Gómez PA Palacio L Enjuanes L. Coronavirus nucleocapsid protein facilitates template switching and is required for efficient transcription. J Virol. (2010) 84:2169–75. doi: 10.1128/JVI.02011-09 PMC2812394 19955314

[8] Liu DX Fung TS Chong KKL Shukla A. Hilgenfeld, R. Accessory proteins of SARS-CoV and other coronaviruses. Antiviral Res. (2014) 109:97–109. doi: 10.1016/j.antiviral.2014.06.013 24995382 PMC7113789

[9] Bian L Liu J Gao F Gao Q He Q Mao Q. Research progress on vaccine efficacy against SARS-CoV-2 variants of concern. Hum Vaccin Immunother. (2022) 18:2057161. doi: 10.1080/21645515.2022.2057161 35438600 PMC9115786

[10] Dejnirattisai W Huo J Zhou D Zahradník J Supasa P Liu C. SARS-CoV-2 Omicron-B. 1. 1. 529 leads to widespread escape from neutralizing antibody responses. Cell. (2022) 185:467–84. doi: 10.1016/j.cell.2021.12.046 PMC8723827 35081335

[11] Alcantara MC Higuchi Y Kirita Y Matoba S Hoshino A. Deep mutational scanning to predict escape from Bebtelovimab in SARS-CoV-2 Omicron subvariants. Vaccines (Basel). (2023) 11:711. doi: 10.3390/vaccines11030711 36992294 PMC10051238

[12] Qu P Evans JP Faraone J Zheng YM Carlin C Anghelina M. Distinct neutralizing antibody escape of SARS-CoV-2 Omicron subvariants BQ. 1, BQ. 1. 1, BA. 4. 6, BF. 7 and BA. 2. 75. 2. bioRxiv. (2022). doi: 10.1101/2022.10.19.512891 PMC9678813 36476380

[13] Dhama K Nainu F Frediansyah A Yatoo MI Mohapatra RK Chakraborty S. Global emerging Omicron variant of SARS-CoV-2: Impacts, challenges and strategies. J Infect Public Health. (2023) 16:4–14. doi: 10.1016/j.jiph.2022.11.024 36446204 PMC9675435

[14] Magazine N Zhang T Wu Y McGee MC Veggiani G Huang W. Mutations and evolution of the SARS-CoV-2 spike protein. Viruses. (2022) 14:640. doi: 10.3390/v14030640 35337047 PMC8949778

[15] Sakurai F Tachibana M Mizuguchi H. Adenovirus vector-based vaccine for infectious diseases. Drug Metab Pharmacokinet. (2022) 42:100432. doi: 10.1016/j.dmpk.2021.100432 34974335 PMC8585960

[16] Xu F Wu S Yi L Peng S Wang F Si W. Safety, mucosal and systemic immunopotency of an aerosolized adenovirus-vectored vaccine against SARS-CoV-2 in rhesus macaques. Emerg Microbes Infect. (2022) 11:438–41. doi: 10.1080/22221751.2022.2030199 PMC8803102 35094672

[17] Wu S Zhong G Zhang J Shuai L Zhang Z Wen Z. A single dose of an adenovirus-vectored vaccine provides protection against SARS-CoV-2 challenge. Nat Commun. (2022) 11:4081. doi: 10.1038/s41467-020-17972-1 PMC7427994 32796842

[18] Li Y Wang L Zhu T Wu S Feng L Cheng P. Establishing China’s national standard for the recombinant adenovirus type 5 vector-based Ebola vaccine (Ad5-EBOV) virus titer. Hum Gene Ther Clin Dev. (2018) 29:226–32. doi: 10.1089/humc.2018.129 30381976

[19] Wang X Xing M Zhang C Yang Y Chi Y Tang X. Neutralizing antibody responses to enterovirus and adenovirus in healthy adults in China. Emerg Microbes Infect. (2014) 3:e30. doi: 10.1038/emi.2014.30 26038738 PMC4051363

[20] Afkhami S D’Agostino MR Zhang A Stacey HD Marzok A Kang A. Respiratory mucosal delivery of next-generation COVID-19 vaccine provides robust protection against both ancestral and variant strains of SARS-CoV-2. Cell. (2022) 185:896–915. doi: 10.1016/j.cell.2022.02.005 35180381 PMC8825346

[21] Li X Wang L Liu J Fang E Liu X Peng Q. Combining intramuscular and intranasal homologous prime-boost with a chimpanzee adenovirus-based COVID-19 vaccine elicits potent humoral and cellular immune responses in mice. Emerg Microbes Infect. (2022) 11:1890–9. doi: 10.1080/22221751.2022.2097479 PMC9331206 35775819

[22] Wang Q Iketani S Li Z Liu L Guo Y Huang Y. Alarming antibody evasion properties of rising SARS-CoV-2 BQ and XBB subvariants. Cell. (2023) 186:279–86.e8. doi: 10.1016/j.cell.2022.12.018 36580913 PMC9747694

[23] Nie J Li Q Wu J Zhao C Hao H Liu H. Quantification of SARS-CoV-2 neutralizing antibody by a pseudotyped virus-based assay. Nat Protoc. (2020) 15:3699–715. doi: 10.1038/s41596-020-0394-5 32978602

[24] Tuekprakhon A Nutalai R Dijokaite-Guraliuc A Zhou D Ginn HM Selvaraj M. Antibody escape of SARS-CoV-2 Omicron BA. 4 and BA. 5 from vaccine and BA. 1 serum. Cell. (2022) 185:2422–33. doi: 10.1016/j.cell.2022.06.005 PMC9181312 35772405

[25] Hachmann NP Miller J Collier ARY Ventura JD Yu J Rowe M. Neutralization escape by SARS-CoV-2 Omicron subvariants BA. 2. 12. 1, BA. 4, and BA. 5. N. Engl J Med. (2022) 387:86–8. doi: 10.1056/NEJMc2206576 PMC9258748 35731894

[26] Harvey WT Carabelli AM Jackson B Gupta RK Thomson EC Harrison EM. SARS-CoV-2 variants, spike mutations and immune escape. Nat Rev Microbiol. (2021) 19:409–24. doi: 10.1038/s41579-021-00573-0 PMC8167834 34075212

[27] Qu P Faraone JN Evans JP Zheng YM Carlin C Anghelina M. Enhanced evasion of neutralizing antibody response by Omicron XBB. 1. 5, CH. 1. 1, and CA. 3. 1 variants. Cell Rep. (2023) 42:112443. doi: 10.1016/j.celrep.2023.112443 37104089 PMC10279473

[28] Kurhade C Zou J Xia H Liu M Chang HC Ren P. Low neutralization of SARS-CoV-2 Omicron BA. 2. 75. 2, BQ. 1. 1 and XBB. 1 by parental mRNA vaccine or a BA. 5 bivalent booster. Nat Med. (2023) 29:344–7. doi: 10.1038/s41591-022-02162-x 36473500

[29] Uraki R Ito M Kiso M Yamayoshi S Iwatsuki-Horimoto K Furusawa Y. Antiviral and bivalent vaccine efficacy against an omicron XBB. 1. 5 isolate. Lancet Infect Dis. (2023) 23:402–3. doi: 10.1016/S1473-3099(23)00070-1 PMC9908083 36773622

[30] Hoffmann M Arora P Nehlmeier I Kempf A Cossmann A Schulz SR. Profound neutralization evasion and augmented host cell entry are hallmarks of the fast-spreading SARS-CoV-2 lineage XBB. 1. 5. Cell Mol Immunol. (2023) 20:419–22. doi: 10.1038/s41423-023-00988-0 PMC9982771 36869193

[31] Miller J Hachmann NP Collier AY Lasrado N Mazurek CR Patio RC. Substantial neutralization escape by SARS-CoV-2 omicron variants BQ. 1. 1 and XBB. 1. N Engl J Med. (2023) 388:662–4. doi: 10.1056/NEJMc2214314 PMC9878581 36652339

[32] Zhang X Chen LL Ip JD Chan WM Hung IFN Yuen KY. Omicron sublineage recombinant XBB evades neutralising antibodies in recipients of BNT162b2 or CoronaVac vaccines. Lancet Microbe. (2023) 4:e131. doi: 10.1016/S2666-5247(22)00335-4 36493789 PMC9725777

[33] Liu L Iketani S Guo Y Chan JFW Wang M Liu L. Striking antibody evasion manifested by the Omicron variant of SARS-CoV-2. Nature. (2022) 602:676–81. doi: 10.1038/s41586-021-04388-0 35016198

[34] Novazzi F Baj A Pasciuta R Genoni A Ferrante FD Tripiciano R. A cluster of SARS-CoV-2 delta variant of concern additionally harboring F490S, Northern Lombardy, Italy. Int J Infect Dis. (2022) 116:271–2. doi: 10.1016/j.ijid.2021.12.362 PMC8731264 34995777

[35] Chen JJ Li LB Peng HH Tian S Ji B Shi C. Neutralization against XBB. 1 and XBB. 1. 5 after omicron subvariants breakthrough infection or reinfection. Lancet Reg Health West Pac. (2023) 33:100759. doi: 10.1016/j.lanwpc.2023.100759 37090240 PMC10114502

[36] Yue C Song W Wang L Jian F Chen X Gao F. ACE2 binding and antibody evasion in enhanced transmissibility of XBB. 1. 5. Lancet Infect Dis. (2023) 23:278–80. doi: 10.1016/S1473-3099(23)00010-5 PMC9897732 36746173

[37] CDC. CDC data tracker (2023). Available online at: https://covid.cdc.gov/covid-data-tracker/#variantproportions.

[38] CDC. COVID-19 Vaccination . Available online at: https://www.cdc.gov/vaccines/covid-19/vaccination-provider-support.html.

[39] He C Alu A Lei H Yang J Hong W Song X. A recombinant spike-XBB. 1. 5 protein vaccine induces broad-spectrum immune responses against XBB. 1. 5-included Omicron variants of SARS-CoV-2. MedComm. (2020) . 2023:e263. doi: 10.1002/mco2.263 PMC10133731 37125241

[40] Moss P. The T cell immune response against SARS-CoV-2. Nat Immunol. (2022) 23:186–93. doi: 10.1038/s41590-021-01122-w 35105982

[41] Tarke A Coelho CH Zhang Z Dan JM Yu ED Methot N. SARS-CoV-2 vaccination induces immunological T cell memory able to cross-recognize variants from Alpha to Omicron. Cell. (2022) 185:847–59. doi: 10.1016/j.cell.2022.01.015 PMC8784649 35139340

